# Exploring well-being and self-care from the heart failure patient perspective for sustainable living

**DOI:** 10.1590/0034-7167-2024-0394

**Published:** 2025-08-22

**Authors:** Julia Gonçalves Escossia Campos, Giulia Gazineo Trindade Assis, Ligia Neres Matos, Marcela Teixeira de Souza, Graciele Oroski Paes, Liana Amorim Correa Trotte, Karla Biancha Silva de Andrade, Marluci Andrade Conceição Stipp

**Affiliations:** I Universidade Federal do Rio de Janeiro. Rio de Janeiro, Rio de Janeiro, Brazil; II Universidade do Estado do Rio de Janeiro. Rio de Janeiro, Rio de Janeiro, Brazil

**Keywords:** Sustainable Development, Heart Failure, Self Care, Health Literacy, Nursing, Indicadores de Desarrollo Sostenible, Insuficiencia Cardíaca, Autocuidado, Alfabetización en Salud, Enfermería

## Abstract

**Objectives:**

to identify well-being and self-care strategies from the perspective of patients with heart failure, highlighting practices that promote a sustainable and healthy life.

**Methods:**

qualitative, descriptive research, carried out between November/2022 and March/2023, through semi-structured interviews, supplemented by records from physical and electronic medical records, with patients treated at the heart failure outpatient service of a university hospital in Rio de Janeiro, analyzed through lexicographic textual analysis, using Descending Hierarchical Classification.

**Results:**

after processing the text *corpus*, six classes were generated, grouped into two distinct thematic chunks (non-pharmacological strategies and practices for health promotion) and (socioeconomic and educational challenges for self-care).

**Final Considerations:**

it is urgent to take actions that increase health literacy among this population, promoting greater autonomy in self-care and improving well-being. Actions in this sense must consider the social and economic determinants and the individual needs of each patient.

## RESUMEN

**Objetivos:**

señalar estrategias de bienestar y autocuidado desde la perspectiva de pacientes con insuficiencia cardíaca, destacando prácticas que promuevan una vida sostenible y saludable.

**Métodos:**

investigación cualitativa, descriptiva, realizado entre noviembre/2022 y marzo/2023, a través de entrevistas semiestructuradas, complementadas con historias clínicas físicas y electrónicas, a pacientes atendidos en el servicio ambulatorio de insuficiencia cardíaca de un hospital universitario de Río de Janeiro, analizados a través de análisis textual lexicográfico, utilizando la Clasificación Jerárquica Descendente.

**Resultados:**

luego del procesamiento del corpus textual, se generaron seis clases agrupadas en dos bloques temáticos diferenciados (estrategias y prácticas no farmacológicas para la promoción de la salud) y (desafíos socioeconómicos y educativos para el autocuidado).

**Consideraciones Finales:**

se necesitan urgentemente acciones que incrementen la alfabetización en salud de esta población, promoviendo una mayor autonomía en el autocuidado y mejorando el bienestar. Las acciones en este sentido deben considerar los determinantes sociales y económicos y las necesidades individuales de cada paciente.

## INTRODUCTION

Heart failure (HF) is a chronic condition that affects millions of people worldwide^(1,2)^, requiring careful management of symptoms and disease progression^(3)^, and ongoing patient involvement in their self-care^(4)^. Several studies have highlighted the importance of effective self-care strategies for HF management, focusing mainly on clinical and practical aspects^(4-8)^. However, there is a significant gap in the literature when it comes to exploring well-being of patients with HF from their own perspectives. This aspect is crucial, as the subjective experience of well-being can directly influence adherence to self-care and, consequently, the quality of life of these individuals.

Furthermore, most studies of patients with HF have focused on hospitalized populations and quantitative approaches^(7,9-11)^. These investigations, although valuable, do not fully capture the complexity and richness of the experiences of patients managing their condition in outpatient settings.

To face the emotional, physical and social challenges imposed by HF, patients adopt several coping strategies, which can influence mortality rates, readmissions, depressive symptoms, psychological well-being, as well as engagement in self-care behaviors^(12,13)^.

In recent decades, the concept of sustainability has attracted worldwide attention and has been widely discussed^(14)^. In 2015, the United Nations established 17 Sustainable Development Goals (SDGs), a global agenda to achieve a more sustainable future by 2030, with Brazil among the signatories^(15,16)^. Although there are multiple definitions of sustainability, it can be defined as the balance of a species and the resources of its environment. Globally, the various definitions of the term have incorporated the concept of improving and maintaining an individual’s well-being over the long term by restoring, adjusting or preserving environmental systems^(17)^.

Thus, health is highlighted as a central component in all SDGs, with the third SDG dedicated to ensuring healthy lives and promoting well-being for all at all ages. Specifically, target 3.4 aims to reduce premature mortality from non-communicable diseases by one third through prevention, treatment and the promotion of mental health and well-being^(15)^.

HF represents a significant burden to healthcare systems globally. Inadequate self-care, including poor adherence to pharmacological and non-pharmacological treatment, contributes to the morbidity and mortality associated with the condition^(18)^. Self-care, as a deliberate human function for maintaining health, is crucial for the well-being of individuals with HF, and is prioritized in multidisciplinary management programs for this condition worldwide^(6,19)^.

Effective management of HF and promotion of self-care are key to achieving target 3.4. Nurses play a vital role in this process by providing comprehensive and coordinated care that not only addresses patients’ clinical needs but also aims to improve their quality of life and overall well-being^(20)^. Therefore, knowing the perspectives of well-being and self-care from patients’ perspective is crucial today, especially in nursing, when it comes to developing practices that value patient-centered care.

## OBJECTIVES

To point out well-being and self-care strategies from the perspective of patients with HF, highlighting practices that promote a sustainable and healthy life.

## METHODS

### Ethical aspects

The study followed ethical and legal guidelines, being approved by the *Universidade Federal do Rio de Janeiro* Research Ethics Committee, respecting all ethical aspects involved in research with human beings, in accordance with the recommendations of the Brazilian National Health Council Resolution 466/2012 and Circular Letter 2/2021. To ensure confidentiality of information, Arabic numerals were used alongside the abbreviation of the word “Subject” (e.g., Subject 1, Subject 2...) to indicate participants’ statements, thus maintaining their anonymity. The Informed Consent Form (ICF) was obtained through a written document, with one copy given to the participant and the other remaining with the researcher.

### Study design

This is a descriptive study using a qualitative approach. The COnsolidated criteria for REport Qualitative research (COREQ)^(21)^ were followed, in accordance with the guide for studies with a qualitative approach.

### Study setting and sample

This study was conducted from November 2022 to March 2023 in an outpatient HF service at a university hospital in Rio de Janeiro.

The study sample was intentionally non-probabilistic, including patients of both sexes, over 18 years of age, at any stage of HF, between NYHA functional classes I and III, undergoing outpatient follow-up. Patients with a score lower than 20 in individuals with no education, and lower than 24 in individuals with education, verified through the Mini Mental State Examination, in addition to patients who, on the day of the scheduled interview, presented acute decompensation of HF, were excluded. Data collection was completed using the theoretical saturation criterion, when the information begins to be repeated, with no more relevant data to discover aspects related to the phenomenon under investigation^(22,23)^.

The Mini Mental State Examination is a tool used to assess cognitive function that encompasses several domains, such as spatial and temporal orientation, immediate and recall memory, calculation skills, language (naming, repetition, comprehension), writing skills, and copying drawings^(24)^.

### Data collection and organization

Data collection was carried out by the first author of the study, a nurse and master’s student with experience and training related to research development. Initially, the researcher approached the setting in order to learn about and integrate with the sector dynamics. Patients were approached before or after outpatient care with an invitation to participate in the research by reading the ICF, answering questions and providing possible clarifications related to the research, and a later date was agreed upon for the collection procedures. In this way, a relationship was established with participants, in which they were aware of the reasons and objectives of the research.

Initially, on the scheduled date, clinical, social and demographic data were collected using a specific instrument with information regarding age, sex, date of birth, self-reported race, address, monthly income, education, telephone, marital status, access to cell phone, television, internet and smartphone, use of alcohol, cigarettes and illicit drugs, date of HF diagnosis, NYHA functional class, presence of other comorbidities and medications used. Moreover, when necessary, it was supplemented with records from physical and electronic medical records. Subsequently, the Mini Mental State Examination was administered to assess patients’ cognitive function. The patients then participated in semi-structured interviews, addressing non-pharmacological practices used by them, those prescribed by healthcare professionals, their sources of knowledge on the subject, and what they considered important to know about their disease and better control of symptoms. The interviews lasted an average of 30 minutes, and were audio recorded and stored on Google Drive^®^.

To organize the data, the interviews were transcribed to form the text *corpus*, and reviewed to correct spelling errors and language defects, and were subsequently validated by the participants. Sociodemographic characteristics, including sex, age, race, marital status, education, time since diagnosis in years, among others, were tabulated in Microsoft Excel^®^.

### Data analysis

The *Interface de R pour les Analyses Multidimensionnelles de Textes et de Questionnaires* (IRaMuTeQ^®^) 0.7 alpha 2 (2020), a free software distributed under the terms of the GNU GPL (v2) license, was used to assist in the *corpus* text analysis. An approach based on the word lemmatization technique was used, which consists of relating words by their root form, disregarding variations such as verb tense, gender, plural and other characteristics of the terms. This approach allowed grouping similar terms and simplifying textual analysis, facilitating the identification of patterns and meanings underlying the analyzed texts (Acauan *et al.*, 2020). Text analysis was performed using Descending Hierarchical Classification, a statistical method proposed by Reinert in 1990, which overcomes the dichotomy between quantitative and qualitative analysis. An index of 75% or more of *corpus* utilization is considered for analysis^(25)^.

Sociodemographic data were analyzed and presented using simple descriptive statistics.

## RESULTS

The research involved the participation of 33 patients, with a predominance of females (51.5%), aged under 60 years (54.5%). The average age of both women and men was approximately 57 years, with 50% of the sample having been diagnosed with HF in less than five years, classified as HF with reduced ejection fraction.

Concerning race/color, most patients self-identified as brown (57.6%), followed by black (27.3%) and white (12.1%). As for the occurrence of other reported comorbidities, there was a prevalence of chronic non-communicable diseases, such as hypertension, with 60.6%, diabetes mellitus, with 30.3%, and dyslipidemia, with 30.3%. Of the patients interviewed, 60.6% had medical prescriptions, evidencing polypharmacy.

Regarding the interviews, after processing the text *corpus*, originated from the transcription, 697 lemmas, 572 active forms and 17 supplementary forms were identified, resulting in 112 text segments, with a utilization of 80.36%, generating six lexical classes. Based on the material analysis, it was possible to group the classes into two distinct thematic chunks: thematic chunk 1, named “non-pharmacological strategies and practices for health promotion”, composed of classes 5, 4, 3 and 6; and thematic chunk 2, titled “socioeconomic and educational challenges for self-care”, which encompassed classes 2 and 1.

Based on participants’ statements, in thematic chunk 1, it was possible to highlight eating habits, factors that contribute to health, compliance with the health team’s guidelines and coping strategies to promote well-being, as demonstrated in Chart 1.

**Chart 1:** Non-pharmacological strategies and practices for health promotion, Rio de Janeiro, Rio de Janeiro, Brazil, 2024

Classes	Statements	Inferences
Class 5: Eating habits	*I take care of my diet, I try to eat fruits, vegetables, rice, beans. I look for natural foods, I avoid fried foods, condiments or processed foods, I like calming teas like chamomile and lemon balm* [...] (Subj. 30; score 134.59). *I drink orange tea, pitanga tea* [...] *I don’t eat salt, little sugar, I use lemon because it’s good for the blood* [...] (Subj. 02; score 74.52).	Concern with healthy eating and adjusting eating habits by avoiding certain foods as well as using healthy alternatives.
Class 4: Factors contributing to health	*I like to walk to help my heart and exercise because my older friends who go to the gym told me I needed to exercise. Now I’m starting to go to the gym to do some light exercise, because my doctor hasn’t recommended it yet* (Subj. 21; score 79.42). *I’m researching my problem, but I haven’t yet discovered the cause, so I’m looking for alternative ways to help professionals find the answer. I seek guidance on healthy eating and dietary restrictions by reading books, the internet, and any other means that can give me a clue about my illness* (Subj. 07; score 55.34).	Adoption of physical exercises, proactive search for information, concern with diagnosis and treatment.
Class 3: Compliance with healthcare team guidelines	*So far, it’s working out well. I don’t do what the internet or other people tell me to do. I follow what the doctor recommends, and I think everyone should follow his instructions too* (Subj. 14; score 70.77). *I was advised to walk, but I can’t because I don’t want to. I was also advised about my diet and water intake, but I still don’t follow it. I drink more than a liter and a half of liquid per day. These were the instructions from the doctor and the nurse. She gave me a spreadsheet with a lot of information, but I can’t put it into practice* (Subj. 33; score 22.83).	Impact of recommendations on daily routine, challenges in implementing guidelines and dependence on professional guidelines versus autonomy.
Class 6: Coping strategies to promote well-being	*I do my activities at home calmly so that I don’t get tired or restless. I watch TV and like to cook to distract myself. If I think too much about my heart problems, I feel sick* (Subj. 29; score 82.79). *If you understand the disease, you start to be careful with your daily activities. I prefer to take it easy so that I don’t get anxious and stressed* (Subj. 08; score 46.38).	Practice recreational activities to reduce stress, integrate self-care into daily life.

The second thematic chunk, described in Chart 2, is composed of socioeconomic aspects that hinder self-care, health literacy and its importance for self-care.

**Chart 2:** Socioeconomic and educational challenges for self-care

Classes	Statements	Inferences
Class 2: Socioeconomic aspects that hinder self-care	*Money is often not enough. I can get my medications through the SUS, through my family clinic; I rarely need to buy medication* (Subj. 26; score 167.25). [...] *I have to buy other medications because I can’t find them through the SUS, which is why I have two jobs to have money. If the person is in a good financial situation, it is easier to treat them* (Subj. 18; score 130.75).	Dependence on public healthcare services, financial impact on the acquisition of medicines, social support and financial aid.
Class 1: Health literacy and its importance for self-care	*It is important to know the real severity of the disease, because then we follow the rules with more determination and without neglecting the treatment* (Subj. 22; score 79.68). *We need to know about the disease to be more careful, to know if I am really well, if they are not deceiving me; communication is important. Knowing what heart failure is, why I have this disease and why I have a big heart* (Subj. 10; score 64.57). *I would like to know what I can do to get better, because sometimes there is no cure or there is no way to operate, but staying comfortable is important* (Subj. 9; score 40.81).	Need to understand the severity of the disease, importance of communication and transparency, guidance of actions based on knowledge.

## DISCUSSION

The survey revealed that most participants were black women, under 60 years old, with hypertension and diabetes, and on polypharmacy. The interviews indicated that well-being strategies include healthy eating, physical exercise and adherence to medical advice, despite the challenges in implementation. Recreational activities positively influence coping with the health condition. However, socioeconomic barriers and lack of knowledge about the disease impair well-being, highlighting the importance of health literacy for better adherence to self-care strategies.

In the context of cardiovascular health, where the challenges posed by heart disease are a global concern, the search for a holistic, humanized and patient-centered approach has been increasingly valued in the health field^(26)^. The predominance of female participants can be explained by the fact that women tend to seek healthcare services more than men, demonstrating greater awareness regarding healthcare^(27)^.

Concerning age group, the study differs from literature on the age profile of patients with HF, which has a higher incidence in the elderly population^(28)^. These data reinforce the need for prevention strategies and ongoing treatment in aging populations, since data from the Mortality Information System^(29)^ from 2021 indicate 492,602 deaths that occurred in hospitals in Brazil, of which 216,844 were considered premature (between 30 and 69 years of age), highlighting the seriousness of the situation, especially in the Southeast, Northeast and South regions of Brazil.

Most patients in this study had two or more comorbidities associated with HF, which is corroborated by another study conducted in northern Brazil, where comorbidities with the same order of prevalence were also identified in patients with HF^(30)^. Moreover, individuals with multimorbidity commonly experience greater use of healthcare, poor coordination of care, and polypharmacy^(31,32)^, as also evidenced in this study.

Considering the complexity of self-care in patients with chronic diseases, the influence of health literacy on quality of life is considered an important determinant of health. A study in southern Spain revealed that those with HF and adequate literacy had greater survival, compared to the low literacy group^(33)^. Therefore, it is observed that low levels of health literacy are linked to negative outcomes, such as lack of adherence to medication, insufficient use of healthcare services, high rates of hospital readmissions^(34)^ and, consequently, a worse sense of well-being.

Well-being is a concept that does not have a specific and universally accepted definition, but it has been identified in the literature, being related to the concept of happiness and good physical and mental health^(35)^. In this study, healthy eating, physical exercise and adherence to medical advice stood out as strategies for maintaining well-being. Entertainment, distraction and humorous behaviors were seen as strategies for coping with the health condition.

Patients express the importance and interest in knowing in depth the nature of their health condition. These statements are in line with the concept of health education for patients and their caregivers, a fundamental component of HF treatment that consists of a process in which the healthcare professional transmits information to patients that can influence their health behavior^(36)^. Nurses play a crucial role in health education, communication and coordination, through individualized care established by mutual agreement between professionals, patients and caregivers, contributing to the empowerment of these individuals in maintaining self-care^(37,38)^.

In this context, self-care is seen as a human function deliberately applied to each person for the purpose of maintaining health, playing a crucial role in achieving the well-being of individuals with HF, being prioritized in multidisciplinary management programs for this condition worldwide^(6,19)^. These strategies not only improve patients’ quality of life, but also have a direct impact on reducing mortality, aligning with SDG target 3.4.

To implement the 17 SDGs, substantial changes in the patterns of societies and economies are necessary. Therefore, the 17 SDGs activate a new phase of shared responsibilities at national, regional and global levels^(39)^. In view of this, when receiving patients, healthcare professionals must be equipped with active listening skills and abilities, in order to identify patients’ needs, respecting their uniqueness, promoting their autonomy and offering comprehensive care that considers the social determinants of health, with an emphasis on improving quality of life^(40)^.

These professionals must have behaviors and attitudes related to the factors that generate harm, based on their skills as health educators. Their guidance should be directed through dialogue between the trinomial composed of the professional, patient and family, in order to resolve doubts and enable self-care, paying attention to the limitations and context in which patients are inserted^(38)^ as well as their individual needs and capabilities^(41)^. This collaboration seeks to increase patient awareness and active participation in relation to their health and well-being, promoting greater autonomy and engagement, contributing to the reduction of premature mortality and the promotion of well-being, as established by SDG 3.4.

Sustainable development is far from simple. The most comprehensive definition of sustainability refers to practices that enable the current population to meet its basic needs without compromising the needs of future generations. The topic of sustainable development has become ubiquitous over the past 30 years, but how to achieve this goal, or even whether it is possible, remains a great question mark^(42)^.

### Study limitations

The study involved patients from a single location, which limits the ability to generalize the results, especially those from different regions, socioeconomic and cultural contexts. Furthermore, its implementation was limited to the outpatient setting. Therefore, wellness practices and access to care may vary significantly across different contexts.

### Contributions to nursing, health, or public policy

Discussing the strategies adopted by patients with HF in managing self-care is crucial for the development of more effective and personalized interventions in the field of nursing. This enables the formulation of effective interventions, through care management, to improve the quality of care and lives of patients^(43,44)^.

This study reinforces the importance of a patient-centered approach, considering all aspects inherent to improving their quality of life. Such an approach highlights the need for nursing practices that go beyond clinical treatment, promoting patients’ full well-being^(45)^. The study also highlights the importance of ongoing patient education about their condition and coping strategies.

By aligning nursing practices with SDG 3.4, which aims to reduce premature mortality from noncommunicable diseases, this study indicates strategies for promoting the population’s health and well-being from the perspective of patients with HF. Evidence-based nursing interventions focused on prevention and management of comorbidities are essential to achieve these goals. Continuous investigation and critical analysis of patient testimonies in research are essential to improve the quality of care provided by healthcare professionals, in order to promote a sustainable and healthy life.

## FINAL CONSIDERATIONS

The study in question, when analyzing self-care and well-being strategies, demonstrates the importance of a patient-centered approach. Even in the face of socioeconomic challenges and lack of knowledge about the disease, the results highlight the need for actions that expand health literacy in this population, promoting greater autonomy in self-care and better health outcomes. The role of nurses as educators and communicators is crucial in this process, and should be guided by active listening, respect for patients’ uniqueness, and the provision of individualized and integrative care.

In this context, health education emerges as an essential tool for empowering patients, contributing to the reduction of premature mortality and promoting well-being, in line with the third SDG. Actions in this sense must consider the social determinants of health and the individual needs of each patient, fostering collaboration between the professional, patient and family.

By investing in health education and promoting self-care, we can contribute to building a fairer and more sustainable future, where everyone has access to quality healthcare and can live with greater well-being.

## Data Availability

The research data are available only upon request.
