# Supplementary material for: Exploring well-being and self-care from the heart failure patient perspective for sustainable living
Source: Rev Bras Enferm. 2025 Aug 22;78(Suppl 1):e20240394. doi: 10.1590/0034-7167-2024-0394 (PMC12380420; doi:10.1590/0034-7167-2024-0394)
Supplement: Translation

# Translation: Explorando bem-estar e autocuidado na perspectiva do paciente com insuficiência cardíaca para uma vida sustentável

**DOI:** 10.1590/0034-7167-2024-0394pt

**Authors:** Julia Gonçalves Escossia Campos, Giulia Gazineo Trindade Assis, Ligia Neres Matos, Marcela Teixeira de Souza, Graciele Oroski Paes, Liana Amorim Correa Trotte, Karla Biancha Silva de Andrade, Marluci Andrade Conceição Stipp

**Affiliations:** I Universidade Federal do Rio de Janeiro. Rio de Janeiro, Rio de Janeiro, Brazil; II Universidade do Estado do Rio de Janeiro. Rio de Janeiro, Rio de Janeiro, Brazil

**Keywords:** Desenvolvimento Sustentável, Insuficiência Cardíaca, Autocuidado, Letramento em Saúde, Enfermagem

## Abstract

**Objetivos:**

apontar as estratégias de bem-estar e autocuidado na perspectiva dos pacientes com insuficiência cardíaca, destacando práticas que promovam uma vida sustentável e saudável.

**Métodos:**

pesquisa qualitativa, de caráter descritivo, realizada entre novembro/2022 e março/2023, por meio de entrevistas semiestruturadas, complementadas pelos registros dos prontuários físicos e eletrônicos, com pacientes atendidos no serviço ambulatorial de insuficiência cardíaca de um hospital universitário do Rio de Janeiro, analisados por meio da análise textual lexicográfica, recorrendo à Classificação Hierárquica Descendente.

**Resultados:**

após o processamento do *corpus* textual, geraram-se seis classes agrupadas em dois blocos temáticos distintos (estratégias e práticas não farmacológicas para a promoção da saúde) e (desafios socioeconômicos e educacionais para o autocuidado).

**Considerações Finais:**

é premente ações que ampliem o letramento em saúde dessa população, promovendo maior autonomia no autocuidado e melhora do bem-estar. Ações nesse sentido devem considerar os determinantes sociais, econômicos e as necessidades individuais de cada paciente.

## INTRODUÇÃO

A insuficiência cardíaca (IC) é uma condição crônica que afeta milhões de pessoas em todo o mundo^(1,2)^, exigindo um gerenciamento cuidadoso dos sintomas e evolução da doença^(3)^, e envolvimento contínuo dos pacientes em seu autocuidado^(4)^. Diversos estudos têm destacado a importância de estratégias de autocuidado eficazes para a gestão da IC, focando principalmente em aspectos clínicos e práticos^(4-8)^. No entanto, há uma lacuna significativa na literatura quando se trata de explorar o bem-estar dos pacientes com IC a partir de suas próprias perspectivas. Este aspecto é crucial, pois a experiência subjetiva do bem-estar pode influenciar diretamente a adesão ao autocuidado e, consequentemente, a qualidade de vida desses indivíduos.

Além disso, a maioria dos estudos sobre pacientes com IC tem se concentrado em populações hospitalizadas e abordagens quantitativas^(7,9-11)^. Essas investigações, embora valiosas, não capturam plenamente a complexidade e a riqueza das experiências de pacientes que gerenciam sua condição em ambientes ambulatoriais.

Para enfrentar os desafios emocionais, físicos e sociais impostos pela IC, os pacientes adotam diversas estratégias de enfrentamento, que pode influenciar taxas de mortalidade, reinternações, sintomas depressivos, bem-estar psicológico, bem como engajamento em comportamentos de autocuidado^(12,13)^.

Nas últimas décadas, o conceito de sustentabilidade tem atraído a atenção mundial, tendo sido amplamente discutido^(14)^. Em 2015, a Organização das Nações Unidas estabeleceu 17 Objetivos de Desenvolvimento Sustentável (ODS), uma agenda global para alcançar um futuro mais sustentável até 2030, com o Brasil entre os signatários^(15,16)^. Embora existam múltiplas definições de sustentabilidade, ela pode ser definida como o equilíbrio de uma espécie e os recursos de seu ambiente. Globalmente, as diversas definições do termo incorporaram o conceito de melhorar e manter o bem-estar de um indivíduo a longo prazo, recuperando, ajustando ou preservando sistemas ambientais^(17)^.

Dessa forma, a saúde é destacada como um componente central em todos os ODS, sendo o seu terceiro objetivo dedicado a assegurar uma vida saudável e promover o bem-estar para todos, em todas as idades. Especificamente, a meta 3.4 visa reduzir em um terço a mortalidade prematura por doenças não transmissíveis através da prevenção, tratamento e promoção da saúde mental e do bem-estar^(15)^.

A IC representa uma carga significativa para os sistemas de saúde globalmente. A inadequação do autocuidado, incluindo a baixa adesão ao tratamento farmacológico e não farmacológico, contribui para a morbimortalidade associada à condição^(18)^. O autocuidado, como uma função humana deliberada para a manutenção da saúde, é crucial para o bem-estar dos indivíduos com IC, sendo priorizado em programas multidisciplinares de gestão dessa condição em todo o mundo^(6,19)^.

A gestão eficaz da IC e a promoção do autocuidado são fundamentais para alcançar a meta 3.4. Os enfermeiros desempenham um papel vital nesse processo, fornecendo cuidados abrangentes e coordenados que não apenas abordam as necessidades clínicas dos pacientes, mas também visam melhorar sua qualidade de vida e bem-estar geral^(20)^. Dessa forma, conhecer as perspectivas de bem-estar e autocuidado sob a ótica do paciente é crucial nos dias atuais e, principalmente, na enfermagem, quando se reporta ao desenvolvimento de práticas que valorizem o cuidado centrado no paciente.

## OBJETIVOS

Apontar as estratégias de bem-estar e autocuidado na perspectiva dos pacientes com IC, destacando práticas que promovam uma vida sustentável e saudável.

## MÉTODOS

### Aspectos éticos

O estudo seguiu as diretrizes éticas e legais, sendo aprovado pelo Comitê de Ética em Pesquisa da Universidade Federal do Rio de Janeiro, respeitando todos os aspectos éticos envolvidos em pesquisas com seres humanos, conforme as recomendações da Resolução do Conselho Nacional de Saúde n° 466/2012 e as recomendações do Ofício Circular nº 2/2021. Para garantia do sigilo das informações, foram utilizados números arábicos junto à abreviação da palavra “Sujeito” (ex., Suj.1, Suj.2...) na indicação dos depoimentos dos participantes, mantendo, dessa forma, seu anonimato. O Termo de Consentimento Livre e Esclarecido (TCLE) foi obtido por meio de documento escrito, sendo uma via entregue ao participante e outra permanecendo com o pesquisador.

### Tipo de estudo

Estudo descritivo, utilizando uma abordagem qualitativa. Para sua realização, foram seguidos os critérios *COnsolidated criteria for REport Qualitative research* (COREQ)^(21)^, de acordo com o guia para estudos com abordagem qualitativa.

### Cenário e amostra do estudo

Este estudo foi conduzido no período de novembro de 2022 a março de 2023, em um serviço ambulatorial de IC de um hospital universitário do Rio de Janeiro.

A amostra do estudo foi não probabilística intencional, sendo ponderados como critérios de inclusão pacientes de ambos os sexos, maiores de 18 anos, em qualquer estágio da IC, entre as classes funcionais I a III da NYHA, em acompanhamento ambulatorial. Os critérios de exclusão foram pacientes com pontuação menor do que 20, em indivíduos sem escolaridade, e menor que 24, em indivíduos com escolaridade, verificados através do Mini Exame do Estado Mental, além de pacientes que, no dia da entrevista agendada, apresentavam quadro de descompensação aguda de IC. A coleta de dados foi finalizada utilizando-se o critério de saturação teórica, quando as informações passam a se repetir, não havendo mais dados relevantes para descobrir aspectos relacionados ao fenômeno em investigação^(22,23)^.

O Mini Exame do Estado Mental é uma ferramenta utilizada para avaliação da função cognitiva que engloba diversos domínios, como orientação espacial e temporal, memória imediata e de evocação, habilidades de cálculo, linguagem (nomeação, repetição, compreensão), habilidades de escrita, e cópia de desenhos^(24)^.

### Coleta e organização dos dados

A coleta de dados foi realizada pela primeira autora do estudo, enfermeira e aluna do curso de mestrado, tendo experiência e treinamento relacionados ao desenvolvimento de pesquisa. Inicialmente, foi realizada uma aproximação da pesquisadora com o cenário, a fim de conhecer e se integrar com a dinâmica do setor. Foram realizados a abordagem ao paciente antes ou após os atendimentos ambulatoriais com o convite para participação da pesquisa com a leitura do TCLE, retirada de dúvidas e possíveis esclarecimentos relacionados à pesquisa, sendo acordada posterior data para realização dos procedimentos de coleta. Dessa forma, foi estabelecida uma relação com os participantes, na qual eles estavam cientes das razões e objetivos da pesquisa.

Inicialmente, na data agendada, foram coletados os dados clínicos, sociais e demográficos por meio de instrumento próprio com informações referentes à idade, sexo, data de nascimento, cor autorreferida, endereço, renda mensal, escolaridade, telefone, estado civil, acesso à celular, televisão, internet e smartphone, uso de álcool, cigarro e drogas ilícitas, data do diagnóstico da IC, classe funcional da NYHA, presença de outras comorbidades e medicamentos utilizados. Além disso, quando necessário, era complementado com os registros dos prontuários físicos e eletrônicos. Posteriormente, foi aplicado o Mini Exame do Estado Mental para avaliar a função cognitiva dos pacientes. Em seguida, os pacientes participaram de entrevistas semiestruturadas, abordando práticas não farmacológicas utilizadas por eles, as prescritas pelos profissionais de saúde, suas fontes de conhecimento sobre o assunto, e o que consideravam importante saber sobre sua doença e melhor controle dos sintomas. A duração das entrevistas teve um período médio de 30 minutos, e foram gravadas em áudio armazenados no *Google Drive* ^®^.

Para a organização dos dados, as entrevistas foram transcritas para a formação do *corpus* textual, sendo revisadas para correção de erros de grafia e vícios de linguagem, sendo, posteriormente, validadas pelos participantes. As características sociodemográficas, incluindo sexo, idade, raça, estado civil, escolaridade, tempo desde o diagnóstico em anos, entre outras, foram tabuladas no *Microsoft Excel* ^®^.

### Análise dos dados

Foi utilizado o *Interface de R pour les Analyses Multidimensionnelles de Textes et de Questionnaires* (IRaMuTeQ^®^) 0.7 alpha 2 (2020), um *software* livre distribuído sob os termos da licença GNU GPL (v2), para auxiliar na análise textual do *corpus*. Foi utilizada uma abordagem baseada na técnica de lematização de palavras, que consiste em relacionar as palavras pela sua forma-raiz, desconsiderando variações como tempo verbal, gênero, plural e outras características dos termos. Essa abordagem permitiu agrupar termos semelhantes e simplificar a análise textual, facilitando a identificação de padrões e significados subjacentes aos textos analisados (Acauan *et al.*, 2020). A análise textual foi realizada utilizando a Classificação Hierárquica Descendente, um método estatístico proposto por Reinert, em 1990, que supera a dicotomia entre análise quantitativa e qualitativa. Considera-se para análise um índice de 75% ou mais de aproveitamento do *corpus* ^(25)^.

Os dados sociodemográficos foram analisados e apresentados através de estatística descritiva simples.

## RESULTADOS

A pesquisa envolveu a participação de 33 pacientes, com predomínio do sexo feminino (51,5%), com idade inferior a 60 anos (54,5%). A idade média, tanto das mulheres quanto dos homens, foi de aproximadamente 57 anos, tendo 50% da amostra seu diagnóstico de IC em período inferior a cinco anos, classificados como IC de fração de ejeção reduzida.

No que tange à raça/cor, a maioria dos pacientes se autodeclarou parda (57,6%), seguida de preta (27,3%) e branca (12,1%). Quanto ao acometimento por outras comorbidades relatadas, nota-se a prevalência de doenças crônicas não transmissíveis, como hipertensão arterial sistêmica, com 60,6%, diabetes mellitus, com 30,3%, e dislipidemia, com 30,3%. Dos pacientes entrevistados, 60,6% possuíam prescrições médicas, evidenciando polifarmácia.

Com relação às entrevistas, após o processamento do *corpus* textual, originado a partir da transcrição, foram identificados 697 lemas, 572 formas ativas e 17 suplementares, resultando em 112 segmentos de texto, com aproveitamento de 80,36%, gerando seis classes lexicais. Com base na análise do material, foi possível agrupar as classes em dois blocos temáticos distintos: o bloco temático 1, nomeado como “estratégias e práticas não farmacológicas para a promoção da saúde”, composto pelas classes 5, 4, 3 e 6; e o bloco temático 2, intitulado como “desafios socioeconômicos e educacionais para o autocuidado”, que englobou as classes 2 e 1.

Pelo relato dos participantes, no bloco temático 1, foi possível evidenciar hábitos alimentares, fatores que contribuem para a saúde, adequação às orientações da equipe de saúde e estratégias de enfrentamento na promoção do bem-estar, conforme demonstrado no Quadro 1.

**Quadro 1:** Estratégias e práticas não farmacológicas para a promoção da saúde, Rio de Janeiro, Rio de Janeiro, Brasil, 2024

Classes	Depoimentos	Inferências
Classe 5: Hábitos alimentares	*Cuidados com a alimentação, procuro comer frutas, legumes, arroz, feijão. Busco alimentos naturais, evito frituras, condimentos ou alimentos industrializados, gosto de chá calmante como camomila e erva cidreira* [...] (Suj. 30; escore 134.59). *Eu tomo um chá de laranja da terra, chá de pitanga* [...] *não como sal, pouco açúcar, uso limão porque é bom para o sangue* [...] (Suj. 02; escore 74.52).	Preocupação com a alimentação saudável e ajuste dos hábitos alimentares por meio da evitação de certos alimentos, além de uso de alternativas saudáveis.
Classe 4: Fatores que contribuem para a saúde	*Gosto de caminhar para ajudar o coração e praticar exercícios por indicação dos meus colegas mais velhos que fazem academia, e me falaram que eu preciso praticar. Agora estou passando a ir à academia para fazer alguns exercícios leves, porque ainda não foi indicado pelo médico* (Suj. 21; escore 79.42). *Pesquiso a respeito do meu problema, ainda não descobri a causa, então busco por meios alternativos ajudar os profissionais a encontrar a resposta. Busco orientações sobre alimentações saudáveis e restrição alimentar pela leitura de livro, internet e todos os meios que possam me dar uma pista da minha enfermidade* (Suj.07; escore 55.34).	Adoção de exercícios físicos, busca proativa por informação, preocupação com diagnóstico e tratamento.
Classe 3: Adequação às orientações da equipe de saúde	*Até o momento, está dando certo, não faço o que a internet ou as pessoas mandam, sigo o que o médico orienta e acho que todo mundo tinha que seguir as orientações também* (Suj. 14; escore 70.77). *Fui orientada a caminhar, mas não consigo por não querer. Também fui orientada quanto à dieta e ingesta hídrica, mesmo assim não sigo, bebo mais de um litro e meio de líquido por dia. Foram orientações do médico e da enfermeira. Ela me deu uma planilha com várias informações, mas não consigo colocar em prática* (Suj. 33; escore 22.83).	Impacto das recomendações na rotina diária, desafios na implementação de orientações e dependência das orientações profissionais *versus* autonomia.
Classe 6: Estratégias de enfrentamento na promoção do bem-estar	*Faço as atividades dentro de casa com calma para não ficar cansada ou parada. Vejo televisão e gosto de cozinhar para me distrair. Se eu fico pensando muito nos problemas do coração, eu passo mal* (Suj. 29; escore 82.79). *Se você entende a doença, começa a tomar cuidado com as atividades no cotidiano. Eu prefiro levar na brincadeira para não ficar ansioso e não me estressar* (Suj. 08; escore 46.38).	Prática de atividades recreativas para redução do estresse, integração do autocuidado na vida cotidiana.

O segundo bloco temático, descrito no Quadro 2, é composto por aspectos socioeconômicos que prejudicam o autocuidado, o letramento em saúde e sua importância para o autocuidado.

**Quadro 2:** Desafios socioeconômicos e educacionais para o autocuidado

Classes	Depoimentos	Inferências
Classe 2: Aspectos socioeconômicos que prejudicam o autocuidado	*O dinheiro muitas vezes não é suficiente. As medicações, eu consigo pegar pelo SUS, pela clínica da família; raras são as vezes que preciso comprar uma medicação* (Suj. 26; escore 167.25). [...] *os outros remédios, eu preciso comprar porque não encontro no SUS, por isso tenho dois trabalhos, para ter dinheiro. Se a pessoa tem uma condição financeira boa, é mais fácil de tratar* (Suj. 18; escore 130.75).	Dependência dos serviços públicos de saúde, impacto financeiro na aquisição de medicamentos, apoio social e ajuda financeira.
Classe 1: Letramento em saúde e sua importância para o autocuidado	*É importante saber a gravidade real da doença, porque, assim, a gente segue a regra com mais determinação e sem descuido no tratamento* (Suj. 22; escore 79.68). *A gente precisa saber sobre a doença para tomar mais cuidado, saber se estou bem mesmo, se não estão me enganando; a comunicação é importante. Saber o que é insuficiência cardíaca, porque eu tenho essa doença e porque eu tenho coração grande* (Suj. 10; escore 64.57). *Gostaria de saber o que posso fazer para melhorar, porque, às vezes, não tem cura ou não tem como operar, mas se manter confortável é importante* (Suj. 9; escore 40.81).	Necessidade de compreender a gravidade da doença, importância da comunicação e transparência, orientação das ações pelo conhecimento.

## DISCUSSÃO

A pesquisa revelou que a maioria dos participantes era mulher negra, abaixo de 60 anos, com hipertensão e diabetes, em polifarmácia. As entrevistas indicaram que estratégias de bem-estar incluem alimentação saudável, exercícios físicos e adesão às orientações médicas, apesar dos desafios na implementação. Atividades recreativas influenciam positivamente o enfrentamento da condição de saúde. Contudo, barreiras socioeconômicas e falta de conhecimento sobre a doença implicam o bem-estar, destacando a importância do letramento em saúde para melhor adesão às estratégias de autocuidado.

No contexto da saúde cardiovascular, onde os desafios impostos pelas doenças cardíacas são uma preocupação global, a busca por uma abordagem holística, humanizada e centrada no paciente tem sido cada vez mais valorizada no campo da saúde^(26)^. A predominância de participantes femininas pode ser explicada pelo fato de que as mulheres tendem a procurar mais os serviços de saúde do que os homens, demonstrando maior conscientização em relação aos cuidados com a saúde^(27)^.

Em relação à faixa etária, o estudo diverge da literatura sobre o perfil de idade dos pacientes com IC, que possui maior incidência na população idosa^(28)^. Esses dados reforçam a necessidade de estratégias de prevenção e tratamento contínuo em populações em envelhecimento, uma vez que dados do Sistema de Informações sobre Mortalidade^(29)^ de 2021 apontam para 492.602 mortes ocorridas em hospitais no Brasil, sendo 216.844 consideradas prematuras (entre 30 e 69 anos), ressaltando a gravidade da situação, especialmente nas regiões Sudeste, Nordeste e Sul do país.

A maioria dos pacientes deste estudo possui duas ou mais comorbidades associadas à IC, o que é corroborado por outro estudo realizado na região Norte do Brasil, onde também foram identificadas comorbidades com igual ordem de prevalência em pacientes com IC^(30)^. Além disso, indivíduos com multimorbidades comumente experimentam maior uso de cuidados de saúde, má coordenação de cuidados e polifarmácia^(31,32)^, conforme também evidenciado neste estudo.

Considerando a complexidade do autocuidado em pacientes com doenças crônicas, a influência do letramento em saúde na qualidade de vida é considerada um importante determinante de saúde. Estudo no sul da Espanha revelou que aqueles com IC e letramento adequado apresentaram maior sobrevida, em comparação com o grupo de baixo letramento^(33)^. Portanto, observa-se que baixos níveis de letramento em saúde estão ligados a desfechos negativos, como falta de adesão à medicação, uso insuficiente dos serviços de saúde, altas taxas de readmissões hospitalares^(34)^ e, consequentemente, pior sensação de bem-estar.

O bem-estar é um conceito que não possui uma definição específica e universalmente aceita, porém tem sido identificado na literatura, estando relacionado ao conceito de felicidade e boa saúde física e mental^(35)^. Neste estudo, destacaram-se como estratégias de manutenção do bem-estar alimentação saudável, exercício físico e adesão às orientações médicas. Já práticas de entretenimento, distração e comportamentos de humor foram vistas como estratégias de enfrentamento à condição de saúde.

Os pacientes expõem a importância e o interesse em conhecer a fundo a natureza da condição de saúde. Essas falas estão em concordância com o conceito da educação em saúde de pacientes e seus cuidadores, um componente fundamental do tratamento da IC que consiste em um processo em que o profissional de saúde transmite aos pacientes informações que podem influenciar seu comportamento de saúde^(36)^. O enfermeiro desempenha um papel crucial na educação em saúde, comunicação e coordenação, por meio de cuidados individualizados estabelecidos em comum acordo entre o profissional, o paciente e os cuidadores, contribuindo para o empoderamento desses indivíduos na manutenção do autocuidado^(37,38)^.

Neste contexto, percebe-se o autocuidado como uma função humana aplicada deliberadamente a cada pessoa para fins da manutenção da saúde, desempenhando um papel crucial para a obtenção de bem-estar dos indivíduos com IC, sendo priorizado nos programas multidisciplinares de gestão desta condição mundialmente^(6,19)^. Essas estratégias não apenas melhoram a qualidade de vida dos pacientes, mas também têm um impacto direto na redução da mortalidade, alinhando-se com a meta 3.4 dos ODS.

Para implementar os 17 ODS, são necessárias modificações substanciais nos padrões das sociedades e economias. Portanto, os 17 ODS ativam uma nova fase de partilha de responsabilidades a nível nacional, regional e global^(39)^. Diante disso, ao receber o paciente, o profissional de saúde deve ser dotado de competências e habilidades de escuta ativa, de forma a identificar as necessidades do paciente, respeitando sua singularidade, promovendo sua autonomia e oferecendo cuidados integrais que considerem os determinantes sociais de saúde, com ênfase na melhoria da qualidade de vida^(40)^.

Esses profissionais devem possuir comportamentos e atitudes relacionados aos fatores que geram agravos, ancorando-se em suas competências como educadores em saúde. Sua orientação deve ser direcionada por meio do diálogo entre o trinômio composto pelo profissional, paciente e família, de forma a solucionar dúvidas e capacitar para o autocuidado, atentando para as limitações e o contexto no qual o paciente está inserido^(38)^, assim como suas necessidades individuais e capacidades^(41)^. Essa colaboração busca aumentar a consciência e a participação ativa do paciente em relação à sua saúde e bem-estar, promovendo maior autonomia e engajamento, contribuindo para a redução da mortalidade prematura e promoção do bem-estar, conforme estabelecido pelo ODS 3.4.

O desenvolvimento sustentável está longe de ser simples. A definição mais abrangente de sustentabilidade refere-se a práticas que permitem à população atual satisfazer as suas necessidades básicas sem comprometer as necessidades das gerações futuras. O tema do desenvolvimento sustentável tornou-se onipresente nos últimos 30 anos, mas como atingir tal objetivo, ou se é mesmo possível, permanece uma grande incógnita^(42)^.

### Limitações do estudo

O estudo envolveu pacientes de uma única localidade, o que limita a capacidade de generalizar os resultados, especialmente aqueles de diferentes regiões, contextos socioeconômicos e culturais. Ademais, a sua realização foi limitada ao contexto ambulatorial. Portanto, as práticas de bem-estar e o acesso aos cuidados podem variar significativamente em diferentes contextos.

### Contribuições para as áreas da enfermagem, saúde, ou políticas públicas

Discutir as estratégias adotadas por pacientes com IC no gerenciamento do autocuidado é crucial para o desenvolvimento de intervenções mais eficazes e personalizadas no campo da enfermagem. Isso possibilita a formulação de intervenções eficazes, através do gerenciamento do cuidado, para aprimorar a qualidade da assistência e a vida dos pacientes^(43,44)^.

Este estudo reforça a importância de uma abordagem centrada no paciente, considerando todos os aspectos inerentes à melhoria de sua qualidade de vida. Tal abordagem destaca a necessidade de práticas de enfermagem que vão além do tratamento clínico, promovendo o bem-estar integral do paciente^(45)^. O estudo também sublinha a relevância da educação contínua dos pacientes sobre sua condição e as estratégias de enfrentamento.

Ao alinhar as práticas de enfermagem com a meta do ODS 3.4, que visa reduzir a mortalidade prematura por doenças não transmissíveis, este estudo indica estratégias de promoção da saúde e bem-estar da população sob a ótica dos pacientes com IC. As intervenções de enfermagem baseadas em evidências, focadas na prevenção e no manejo de comorbidades, são essenciais para atingir esses objetivos. A contínua investigação e análise crítica dos depoimentos dos pacientes em pesquisas são essenciais para a melhoria da qualidade do cuidado ofertado pelos profissionais de saúde, de forma a promover uma vida sustentável e saudável.

## CONSIDERAÇÕES FINAIS

O estudo em questão, ao analisar as estratégias de autocuidado e bem-estar, demonstra a importância de uma abordagem centrada no paciente. Mesmo diante de desafios socioeconômicos e falta de conhecimento sobre a doença, os resultados evidenciam a necessidade de ações que ampliem o letramento em saúde dessa população, promovendo maior autonomia no autocuidado e melhores desfechos em saúde. O papel do enfermeiro como educador e comunicador é crucial nesse processo, devendo ser pautado pela escuta ativa, respeito à singularidade do paciente e oferta de cuidados individualizados e integrativos.

Nesse contexto, a educação em saúde surge como ferramenta essencial para o empoderamento de pacientes, contribuindo para a redução da mortalidade prematura e promoção do bem-estar, alinhado ao terceiro ODS. Ações nesse sentido devem considerar os determinantes sociais de saúde e as necessidades individuais de cada paciente, fomentando a colaboração entre o profissional, paciente e família.

Ao investir na educação em saúde e na promoção do autocuidado, podemos contribuir para a construção de um futuro mais justo e sustentável, onde todos tenham acesso à saúde de qualidade e possam viver com mais bem-estar.

## Data Availability

Os dados de pesquisa só estão disponíveis mediante solicitação.
